# State-Specific Rates of Primary and Secondary Syphilis Among Men Who Have Sex with Men — United States, 2015

**DOI:** 10.15585/mmwr.mm6613a1

**Published:** 2017-04-07

**Authors:** Alex de Voux, Sarah Kidd, Jeremy A. Grey, Eli S. Rosenberg, Thomas L. Gift, Hillard Weinstock, Kyle T. Bernstein

**Affiliations:** ^1^Epidemic Intelligence Service, CDC; ^2^Division of STD Prevention, National Center for HIV/AIDS, Viral Hepatitis, STD, and TB Prevention, CDC; ^3^Department of Epidemiology, Rollins School of Public Health, Emory University, Atlanta, Georgia.

In 2015, the rate of reported primary and secondary syphilis in the United States was 7.5 cases per 100,000 population, nearly four times the previous lowest documented rate of 2.1 in 2000 (*1*). In 2015, 81.7% of male primary and secondary syphilis cases with information on the sex of the sex partner were among gay, bisexual, and other men who have sex with men (collectively referred to as MSM) (*1*). These data suggest a disproportionate incidence of disease among MSM. However, attempts to quantify this disparity have been hindered by limited data on the size of the MSM population at the state level. To produce the first estimates of state-specific rates of primary and secondary syphilis among MSM, CDC used MSM population estimates based on a new methodology (*2*) and primary and secondary syphilis case counts reported in 2015 to the National Notifiable Diseases Surveillance System. Among 44 states reporting information on the sex of sex partners for ≥70% of male cases, the overall rate of primary and secondary syphilis among all men (aged ≥18 years) in the United States in 2015 was 17.5 per 100,000, compared with 309.0 among MSM and 2.9 among men who reported sex with women only. The overall rate of primary and secondary syphilis among MSM was 106.0 times the rate among men who have sex with women only and 167.5 times the rate among women.* These data highlight the disproportionate impact of syphilis among MSM and underscore the need for innovative and targeted syphilis prevention measures at the state and local level, especially among MSM. It is important that health care providers recognize the signs and symptoms of syphilis, screen sexually active MSM for syphilis at least annually, and provide timely treatment according to national sexually transmitted diseases treatment guidelines (*3*).

Case reports of primary and secondary syphilis cases for MSM, men who have sex with women only, and women were obtained from national data reported regularly by all states for 2015. These data include limited demographic and clinical information, including the sex of sex partners. Population estimates of the number of MSM by state were obtained using new methodology that makes use of census and population-based survey data (*2*). To estimate the MSM population size, the estimated percentage of MSM among men was adjusted (*4*) according to each U.S. county’s percentage of households with a male head and a male partner, obtained from American Community Survey summary data and urban-rural classification (large central metropolitan, large fringe metropolitan, medium or small metropolitan, or nonmetropolitan or rural) from the National Center for Health Statistics (*4*). The county’s percentage of MSM was adjusted according to the ratio of its percentage of male same-sex households to the overall percentage among all counties at the same urban-rural classification, which was then multiplied by the number of men in the county to achieve the estimated MSM population size. This final number was then scaled to equal 3.9% of the adult male population, based on a prior national MSM estimate (*5*).

To optimize stability of the estimates, the analysis was limited to the 44 states that included sex of sex partner in ≥70% of male primary and secondary syphilis case reports for 2015. The 70% threshold represented the best balance between including male cases of primary and secondary syphilis while gathering the most complete epidemiologic data for those cases. State-specific rates of primary and secondary syphilis among MSM were compared with rates of primary and secondary syphilis among men who have sex with women only and also among women (cases in men with unknown sex of sex partner were excluded from this analysis). Rate ratios were calculated as 1) the rate of primary and secondary syphilis among MSM divided by the rate among men who have sex with women only and 2) the rate among MSM divided by the rate among women.^†^

Primary and secondary syphilis cases in the 44 states included in the analysis accounted for 83.4% of all 23,872 reported primary and secondary syphilis cases in the United States in 2015. Among the reported primary and secondary syphilis cases among men and women in these 44 states in 2015, 12,118 (60.8%) were among MSM, including 10,942 (54.9%) among men who had sex with men only and 1,176 (5.9%) cases among men who had sex with both men and women.

Among the 44 states, the overall rates of primary and secondary syphilis in 2015 among all men, MSM, men who have sex with women only, and women were 17.5, 309.0, 2.9, and 1.8 cases per 100,000 population, respectively. State-specific rates among MSM ranged from 73.1 per 100,000 population (Alaska) to 748.3 (North Carolina) (Table 1). The overall U.S. rate of primary and secondary syphilis among MSM was 106.0 times the rate among men who have sex with women only, with state-specific rate ratios ranging from 39.2 (Minnesota) to 342.1 (Hawaii). The overall rate of primary and secondary syphilis among MSM was 167.5 times the rate among women, with state-specific rate ratios ranging from 63.7 (Louisiana) to 2,140.3 (Hawaii).

**TABLE 1:** Rates and rate ratios for primary and secondary syphilis among men who have sex with men (MSM), among men who have sex with women only, and among women, by state and overall — United States, 2015*

State^†^	MSM		Rate of primary and secondary syphilis per 100,000 population			Rate ratio^§^	
	Estimated no. in population	% of all men	MSM	Men who have sex with women only	Women	MSM compared with men who have sex with women only	MSM compared with women
**Overall**	**3,921,515**	**3.8**	**309.0**	**2.9**	**1.8**	**106.0**	**167.5**
Alabama	41,822	2.3	320.4	2.4	1.9	131.5	169.4
Alaska	5,469	1.9	73.1	1.1	0.4	67.8	189.5
Arizona	112,102	4.5	385.4	3.3	1.7	116.1	222.0
Arkansas	19,101	1.7	314.1	3.4	2.2	92.9	140.6
California	796,926	5.5	332.2	3.9	3.1	85.8	108.0
Colorado	74,742	3.6	248.9	1.2	0.2	205.5	1,023.7
Connecticut	43,542	3.2	112.5	0.7	1.0	162.7	117.6
Florida	351,797	4.6	370.1	4.5	2.4	82.7	152.3
Hawaii	15,707	2.8	388.4	1.1	0.2	342.1	2,140.3
Idaho	9,979	1.7	320.7	2.4	1.3	131.0	242.7
Illinois	199,075	4.1	311.9	2.5	1.5	124.6	203.8
Indiana	72,413	3.0	290.0	1.5	1.1	193.3	266.6
Iowa	20,924	1.8	219.8	1.0	0.4	226.7	531.7
Kansas	21,906	2.0	228.2	1.3	1.4	169.6	168.1
Kentucky	47,576	2.9	159.7	1.9	1.3	84.5	126.8
Louisiana	43,204	2.5	601.8	8.4	9.5	71.9	63.7
Maine	14,375	2.8	118.3	0.4	1.1	295.3	108.9
Maryland	83,668	3.8	325.1	4.5	2.4	72.0	137.9
Massachusetts	110,254	4.3	278.4	1.1	0.9	247.3	324.2
Michigan	116,354	3.1	233.8	1.4	0.8	163.8	280.2
Minnesota	82,510	4.0	147.9	3.8	1.7	39.2	87.0
Mississippi	20,184	1.9	658.9	4.1	2.6	161.0	251.3
Missouri	72,875	3.2	204.5	3.8	2.2	53.9	93.0
Montana	6,800	1.7	132.4	0.5	0.0	254.1	—^¶^
Nevada	51,990	4.8	398.2	4.9	1.8	81.3	216.6
New Hampshire	13,868	2.7	187.5	1.2	0.6	155.3	337.8
New Jersey	136,271	4.1	152.6	1.3	0.7	117.2	219.3
New Mexico	18,675	2.4	428.4	2.5	1.4	169.2	314.0
North Carolina	105,707	2.9	748.3	5.3	2.7	140.0	278.2
North Dakota	4,840	1.7	165.3	1.1	0.0	150.4	—
Ohio	146,033	3.4	214.3	2.9	1.4	73.3	157.5
Oklahoma	37,006	2.6	418.9	2.3	1.4	185.4	297.6
Oregon	60,932	4.0	313.5	2.8	2.2	111.9	142.1
Pennsylvania	162,848	3.3	256.1	1.6	0.8	159.3	310.5
Rhode Island	24,745	6.1	226.3	2.7	0.9	84.6	248.9
South Carolina	35,388	2.0	536.9	2.9	1.7	187.8	307.9
South Dakota	4,937	1.5	405.1	2.6	2.2	156.2	186.2
Tennessee	73,460	3.0	325.3	2.8	0.9	115.4	371.3
Texas	378,310	3.9	289.4	3.2	2.2	90.1	133.9
Utah	33,898	3.3	132.8	0.5	0.2	251.1	679.2
Vermont	7,142	2.9	126.0	0.0	0.0	—	—
Virginia	115,515	3.7	210.4	1.5	0.5	138.3	436.0
Washington	113,504	4.2	306.6	1.9	1.1	160.6	290.6
West Virginia	13,141	1.8	197.9	2.3	1.2	87.2	165.0

* Data based on 2015 cases reported to CDC by June 8, 2016.

^†^ To optimize stability of the estimates, the analysis was limited to the 44 states that included sex of sex partner in ≥70% of male primary and secondary syphilis case reports for 2015.

^§^ Rate ratios were calculated as 1) the rate of primary and secondary syphilis among MSM divided by the rate among men who have sex with women only and 2) the rate among MSM divided by the rate among women. In this report “women” is used to describe both females aged ≥18 years (used for calculating rates for women), and females of unknown ages (used for calculating rates for men who had sex with women only). Rate ratios were rounded to tenths.

^¶^ Montana, North Dakota, and Vermont had no cases of primary and secondary syphilis reported among women for 2015, resulting in an undefined rate ratio comparing MSM with women. Vermont had no cases of primary and secondary syphilis reported among men who had sex with women only in 2015, resulting in an undefined rate ratio comparing MSM with men who have sex with women only.^.^

Rates of primary and secondary syphilis among MSM varied by U.S. Census region and by state, with the highest rates in the South and West. Four of the five states with the highest primary and secondary syphilis rates among MSM were southern states (Louisiana, Mississippi, North Carolina, and South Carolina) (Table 2). Among states with the 10 highest rates of primary and secondary syphilis in the United States in 2015 (*1*), five states (Arizona, Louisiana, Mississippi, Nevada, and North Carolina) also ranked among the top 10 states with the highest rates of primary and secondary syphilis among MSM (Table 2).

**TABLE 2:** States ranked from highest to lowest, by rates of primary and secondary syphilis cases overall and among men who have sex with men (MSM) and men who have sex with women only, and by rate ratios comparing the rates for MSM with the rates for men who have sex with women only and the rates for women — United States, 2015*

Rank^†^	Rate of primary and secondary syphilis per 100,000 population			Rate ratio^§^	
	Overall primary and secondary syphilis	Primary and secondary syphilis among MSM	Primary and secondary syphilis among men who have sex with women only	MSM compared with men who have sex with women only	MSM compared with women
1	Louisiana	North Carolina	Louisiana	Hawaii	Hawaii
2	California	Mississippi	North Carolina	Maine	Colorado
3	North Carolina	Louisiana	Nevada	Montana	Utah
4	Nevada	South Carolina	Maryland	Utah	Iowa
5	Florida	New Mexico	Florida	Massachusetts	Virginia
6	Arizona	Oklahoma	Mississippi	Iowa	Tennessee
7	Oregon	South Dakota	California	Colorado	New Hampshire
8	Maryland	Nevada	Missouri	Indiana	Massachusetts
9	Illinois	Hawaii	Minnesota	South Carolina	New Mexico
10	Mississippi	Arizona	Arkansas	Oklahoma	Pennsylvania
11	Rhode Island	Florida	Arizona	Kansas	South Carolina
12	Hawaii	California	Texas	New Mexico	Oklahoma
13	Washington	Tennessee	Ohio	Michigan	Washington
14	Texas	Maryland	South Carolina	Connecticut	Michigan
15	Massachusetts	Idaho	Tennessee	Mississippi	North Carolina
16	South Carolina	Alabama	Oregon	Washington	Indiana
17	Alabama	Arkansas	Rhode Island	Pennsylvania	Mississippi
18	New Mexico	Oregon	South Dakota	South Dakota	Rhode Island
19	Oklahoma	Illinois	New Mexico	New Hampshire	Idaho
20	Tennessee	Washington	Illinois	North Dakota	Arizona
21	Pennsylvania	Indiana	Idaho	North Carolina	New Jersey
22	Missouri	Texas	Alabama	Virginia	Nevada
23	Ohio	Massachusetts	West Virginia	Alabama	Illinois
24	Colorado	Pennsylvania	Oklahoma	Idaho	Alaska
25	South Dakota	Colorado	Washington	Illinois	South Dakota
26	Arkansas	Michigan	Kentucky	New Jersey	Alabama
27	Minnesota	Kansas	Pennsylvania	Arizona	Kansas
28	Indiana	Rhode Island	Virginia	Tennessee	West Virginia
29	New Jersey	Iowa	Indiana	Oregon	Ohio
30	Michigan	Ohio	Michigan	Arkansas	Florida
31	Virginia	Virginia	Kansas	Texas	Oregon
32	Idaho	Missouri	New Jersey	West Virginia	Arkansas
33	Kentucky	West Virginia	Colorado	California	Maryland
34	New Hampshire	New Hampshire	New Hampshire	Rhode Island	Texas
35	Kansas	North Dakota	Hawaii	Kentucky	Kentucky
36	West Virginia	Kentucky	Massachusetts	Florida	Connecticut
37	Connecticut	New Jersey	North Dakota	Nevada	Maine
38	Iowa	Minnesota	Alaska	Ohio	California
39	Utah	Utah	Iowa	Maryland	Missouri
40	Maine	Montana	Connecticut	Louisiana	Minnesota
41	North Dakota	Vermont	Utah	Alaska	Louisiana
42	Vermont	Maine	Montana	Missouri	—^¶^
43	Montana	Connecticut	Maine	Minnesota	—
44	Alaska	Alaska	Vermont	—	—

* Data based on 2015 cases reported to CDC by June 8, 2016.

^†^ To optimize stability of the estimates, the analysis was limited to the 44 states that included the sex of sex partners in ≥70% of male primary and secondary syphilis case reports for 2015.

^§^ Rate ratios were calculated as 1) the rate of primary and secondary syphilis among MSM divided by the rate among men who have sex with women only and 2) the rate among MSM divided by the rate among women. In this report “women” is used to describe both females aged ≥18 years (used for calculating rates for women), and females of unknown ages (used for calculating rates for men who had sex with women only).

^¶^ Montana, North Dakota, and Vermont had no cases of primary and secondary syphilis reported among women for 2015, resulting in an undefined rate ratio comparing MSM with women. Vermont had no cases of primary and secondary syphilis reported among men who had sex with women only in 2015, resulting in an undefined rate ratio comparing MSM with men who have sex with women only.^.^

## Discussion

These are the first state-specific rates of primary and secondary syphilis reported for MSM in the United States. The lowest state-specific MSM primary and secondary syphilis rate (73.1 in Alaska) exceeded the highest overall U.S. primary and secondary syphilis rate (70.9), which was observed in 1946. In every state, the incidence of reported syphilis among MSM was higher than the incidence among men who have sex with women only, with rate ratios ranging from 39.2 to 342.1. These data support CDC’s earlier findings using national population size estimates, which highlighted national disparities in syphilis incidence. In the earlier findings, the rate of syphilis incidence among MSM was estimated to be 154 per 100,000 population, compared with 2.2 per 100,000 among other men, resulting in a rate ratio of 71 (*5*), in comparison to the estimate of 106.0 in the current analysis. However, the previous findings were limited in their applicability to state or local areas because the percentage of adult males who are MSM varies widely among states.

Although state-specific incidence rates varied, even in low incidence states (e.g., North Dakota), syphilis rates among MSM were higher than those among men who have sex with women only. The geographic variation highlights the importance of these data for state and local health departments, which can use these data to better understand their local syphilis epidemiology and target resources and interventions to address disparities between MSM and other population groups. The comparison of state-specific rates also highlights the high disease incidence in the South. Four of the five states with the highest primary and secondary syphilis incidence rates among MSM in 2015 were southern states. The estimates of state-specific rates among men who have sex with women only, although lower than those among MSM, also have implications for the rates of syphilis among women. Trends in congenital syphilis tend to follow trends in the incidence of primary and secondary syphilis among women of reproductive age, which has been increasing recently (*6*). Congenital syphilis can result in serious health consequences in infants (*6*). Although CDC is limited by its data usage agreement with the Council of State and Territorial Epidemiologists to conduct data analysis at the state level (*7*), further analyses at the county level by state and local health jurisdictions could be helpful to inform public health action by elucidating geographic disparities in greater detail.

The findings in this report are subject to at least four limitations. First, analyses were restricted to states where the sex of sex partners (male, female, or both) was reported for ≥70% of male cases of primary and secondary syphilis cases during 2015. Although 83.4% of all reported primary and secondary syphilis reported in the United States during 2015 were included, these jurisdictions might not be representative of all persons who receive a diagnosis of primary and secondary syphilis. Second, the denominators used in calculating the rates of primary and secondary syphilis were estimates of the number of MSM in each state, based on the reporting of same-sex households in the American Community Survey; underreporting of same-sex households could result in an underestimation of the MSM population and an overestimation of primary and secondary syphilis rates. Third, cases of syphilis in men for whom the sex of sex partners was unknown were excluded in calculations for both MSM and men who have sex with women only. If MSM are more likely to underreport the sex of their sex partner, this might result in an underestimation of the rate of syphilis among MSM and consequent rate ratio when comparing syphilis rates among MSM and men who have sex with women only. Improving the quality of case report data regarding sex of sex partner information could increase the awareness of public health officials regarding the characteristics of syphilis within their communities. Finally, primary and secondary syphilis case report data likely underestimate the actual number of incident syphilis infections in the United States because not all infections are diagnosed and reported (*8*).

Despite these limitations, these findings are consistent with previous reports that showed pronounced disparities in primary and secondary syphilis rates between MSM and men who have sex with women only (*5*), and the use of state-specific MSM population sizes and primary and secondary syphilis case counts permits comparison of primary and secondary syphilis rates by state. Rates among MSM compared with men who have sex with women only were higher in every state, but state-specific data suggested that certain states might have a greater need for syphilis prevention. Because MSM represent the majority of all primary and secondary syphilis cases, the success of syphilis prevention programs is contingent upon addressing the high rates of syphilis among MSM. It is important that both private and public health care providers 1) recognize the signs and symptoms of syphilis, 2) conduct a comprehensive sexual history, 3) screen all sexually active MSM for syphilis at least annually, and 4) provide timely treatment according to national sexually transmitted diseases treatment guidelines (*3*). Part of this sexual history includes eliciting information on sexual practices and the sex of patients’ sex partners.^§^

**Summary**

**What is already known about this topic?**

Syphilis rates in the United States have been steadily increasing since 2001, and gay, bisexual, and other men who have sex with men (collectively referred to as MSM) represent a disproportionate number of cases. In the absence of reliable, state-specific denominators it has been difficult to estimate state-specific rates and rate ratios to accurately measure the geographic variation and disparity.

**What is added by this report?**

State-specific rate ratios comparing the rate of syphilis among MSM with the rate among men reporting sex with women only ranged from 39.2 (Minnesota) to 342.1 (Hawaii); overall, MSM had a rate of primary and secondary syphilis 106.0 times the rate among men who reported sex with women only.

**What are the implications for public health practice?**

These state-specific rates further highlight the disproportionate impact of syphilis among MSM. Providers should screen sexually active MSM for syphilis at least annually and provide timely treatment according to national sexually transmitted diseases treatment guidelines.

## Footnotes

* In this report “women” is used to describe both females aged ≥18 years (used for calculating rates for women) and females of unknown ages (used for calculating rates for men who had sex with women only).

† Rate ratios were rounded to tenths.

§ https://www.cdc.gov/STD/treatment/SexualHistory.pdf.
